# Timing of ENDS Uptake by Sexual Orientation among Adolescents and Young Adults in Urban Texas

**DOI:** 10.1093/ntr/ntab181

**Published:** 2021-12-04

**Authors:** Irene Tami-Maury, Baojiang Chen, Aslesha Sumbe, Melissa B Harrell

**Affiliations:** Department of Epidemiology, Human Genetics, and Environmental Sciences, University of Texas Health Science Center at Houston, Houston, TX, USA

## Abstract

**Introduction:**

Early-onset of Electronic Nicotine Delivering Systems (ENDS) use puts users at higher risk of developing a regular ENDS use pattern and/or transitioning to combusted tobacco products. Previous studies on ENDS use among adolescents have not considered sexual orientation as a fluid trait that can change over time. Our objective was to evaluate whether ENDS initiation differed by sexual orientation in a longitudinal, population-based cohort of adolescents transitioning into young adulthood in Texas.

**Methods:**

Sample (*n* = 1712) was drawn from the Texas Adolescent Tobacco and Marketing Surveillance System (waves 5–11) and stratified into three groups, representing sexual orientation: (1) respondents who reported being heterosexuals at each wave (straight), (2) those who consistently self-identified as lesbian, gay or bisexual individuals (LGB), and (3) subjects who reported sexual orientation mobility across waves (mobile). Nonparametric models for interval-censored data were used to estimate the cumulative distribution of age at ENDS initiation by sexual orientation group. Cox models for interval-censored data were used to evaluate whether ENDS initiation varied by sexual orientation group after adjusting for sex assigned at birth, race/ethnicity, cohort, and socioeconomic status.

**Results:**

Compared to Straight adolescents, the risk of earlier-onset of ENDS use was higher among mobile individuals (HR = 1.43, 95% CI: 1.12 to 1.83) and LGB individuals (HR = 1.49, 95% CI: 1.13 to 1.98), respectively, after adjusting for sociodemographic risk factors. Differences between Straight adolescents and LGB/mobile individuals became more pronounced with increasing age.

**Conclusion:**

Analyzing sexual mobility overtime is necessary for understanding the risk associated with youth ENDS initiation and subsequent use.

**Implications:**

Future research should use more accurate sexual orientation assessments to explore further the relationship between sexual orientation mobility and early-onset Electronic Nicotine Delivering Systems (ENDS) use. Understanding the implications of sexual orientation mobility on ENDS initiation will be critical for developing inclusive public health programs aimed at preventing or delaying ENDS use and for providing practical recommendations at state and local levels.

## Introduction

As the consumption of combustible cigarettes among youth in the United States (US) has declined over the decade, the use of Electronic Nicotine Delivering Systems (ENDS) or electronic cigarettes (e-cigarettes) has seen a drastic increase. Since 2014, ENDS have been the most commonly reported tobacco product currently used by youth; 16.6% and 34.5% of 8th and 12th graders reported past 30-day vaping in 2020, respectively.^1^ Apart from being detrimental to adolescent health due to the long-term neurobiological alterations associated with nicotine exposure,^2,3^ ENDS have also been associated with e-cigarette or vaping product use associated lung injury (EVALI) outbreak, which began in 2019.^4^ The clinical variability among adolescents diagnosed with EVALI can range from minimal or no supplemental oxygenation to invasive mechanical ventilation, admission to the intensive care unit, and death.^5^ While 82% of patients hospitalized with EVALI cases reported by the Centers for Disease Control and Prevention (CDC)^4^ were associated with tetrahydrocannabinol (THC)-containing product use, 57% of these patients reported using any nicotine-containing products, which cannot rule out the potential contribution of non-THC–containing products, such nicotine, in EVALI cases.

The overall consumption of tobacco products is disproportionately high among sexual and gender minority (SGM) youth.^6–9^ Tobacco use serves as a coping mechanism for sexual minority individuals (i.e., those with non-heterosexual sexual orientations), as well as gender minority individuals (i.e., those whose genders do not match their assigned sex at birth) to contend with stressors like stigma, discrimination, prejudice, violence, internalized homophobia, identity concealment, and fear of identity disclosure.^10,11^

Three in 10 lesbian, gay male, and bisexual (LGB) youth report current ENDS use, which is 25% higher than their heterosexual counterparts.^12^ In a cross-sectional analysis of Texas college students’ data, SGM individuals reported earlier ENDS initiation than their heterosexual counterparts.^13^ However, in most of these ENDS research efforts, sexual orientation is assumed as a rigid trait, while in truth, it can be fluid and subject to changes at any point in a person’s lifetime. The Kinsey Reports,^14,15^ and other research efforts,^16–23^ have provided substantial evidence that sexual orientation can be considered a continuum rather than a fixed category. This may be particularly relevant to adolescence and young adulthood when developmental changes across a wide range of behaviors occur quickly.^24^ Hence, assessing self-reported sexual orientation at one point in time can lead to misclassification bias and incorrect research conclusions, as it fails to consider this potential for mobility or fluidity, particularly during adolescent years.^20^ Since the late-1990s, a great deal of research has emerged about the conscious process of sexual orientation questioning and development across adolescence and young adulthood.^25–27^ Despite some increase in research raising awareness that sexual orientation is not a dichotomous, permanent trait,^17,23,26,28–30^ long-term mobility remains under-investigated, and its association with ENDS use remains unacknowledged.

The Texas Adolescent Tobacco and Marketing Surveillance System (TATAMS) is a study of ENDS use among a population-based cohort of adolescents (*n* = 3907; *N* = 460 069) living in major metropolitan areas of Texas (Houston, San Antonio, Dallas, Austin, and Fort Worth). Since 2014, data on ENDS use behaviors have been collected every 6 months, and in 2016, measures specific to sexual orientation were added.^31^ Because of the longitudinal nature of this study with repeated assessments, it is possible to determine the stability or shifting of sexual orientation among enrollees, as well as how this relates to the onset of ENDS use. In addition, examining the age of initiation of ENDS use will be vital to inform the development of preventive interventions, as early-onset ENDS users are at higher risk of developing a regular use pattern and/or transitioning to combusted tobacco products.^32^ To our knowledge, this study is the first research effort examining sexual orientation mobility and ENDS initiation among a longitudinal cohort of school-aged adolescents transitioning into young adulthood.

## Method

### Study Design and Participants

The study is a secondary analysis of longitudinal data from the Texas Adolescent Tobacco and Marketing Surveillance system (TATAMS). In 2014–2015, TATAMS recruited three population-based cohorts of adolescents (*n* = 3907; *N* = 460 069) in major metropolitan areas of Texas (Houston, San Antonio, Dallas, Austin, and Fort Worth) using a complex, multistage probability design.^31^ Participants were in 8th, 10th, and 12th grades at Wave 5; then in 12th grade, 1, and 3 years post-high school at Wave 11. Retention rates across these surveys ranged from 70% to 86% and did not vary by cohort. Additional description of this methodology has been published elsewhere.^31^ A web-based survey was administered to all study participants at baseline and every six months thereafter. Active, informed consent was obtained from parents and students (minors were asked for their assent), approved by the Center for the Protection of Human Subjects at the University of Texas Health Science Center at Houston (HSC-SPH-13-0377).

This analysis only uses data from Wave 5 (Fall, 2016) through Wave 11 (Spring, 2020), as Wave 5 was the first survey that included questions specific to assessing sexual orientation. The eligible sample for this analysis included any participant who had no missing data on birthdates or survey dates were never ENDS users at baseline (i.e., Wave 5 or later waves) and reported their sexual orientation at least in two waves (*n* = 1712).

### Measures

#### ENDS Initiation

Our dependent variable was ENDS initiation. At each Wave, participants were asked “Have you EVER used an electronic cigarette, vape pen, or e-hookah, even one or two puffs? Marijuana does not count.” The question was preceded by a descriptive text that provided examples of electronic cigarettes, including JUUL, along with a picture. ENDS initiation was defined among baseline never ENDS users who responded “Yes” to the question above at a subsequent wave (Wave 6 to Wave 11), independently of other tobacco product use.

#### Age of ENDS Initiation

The exact date of ENDS initiation for each subject was not available due to the study design. However, a lower and upper age (by weeks) bound were observed, between which the ever ENDS initiation may occur, leading to an interval-censored outcome. The lower bound was the age at the last wave that the subject reported never ENDS use, and the upper bound was the first wave that the subject reported ever ENDS use. For those who were never users at the last wave, the upper bound was set to infinity.

#### Sexual Orientation

Our primary independent variable was a single question on sexual orientation: “Do you consider yourself to be…?” (a) “Straight;” (b) “Lesbian, gay, or bisexual”; (c) “I don’t identify with either of these;” (d) “I don’t know;” or (e) “I would prefer not to say.” This question was asked on all surveys, except Wave 7 and Wave 8. Combining answers to this question across Waves, we stratified the study sample into mutually-exclusive groups: (1) Straight: those who reported “straight” at each wave (*n* = 1381), (2) Lesbian, Gay, Bisexual (LGB): those who reported “lesbian, gay, or bisexual” at each wave (*n* = 139), and (3) mobile: those who reported a combination of “straight” and/or “LGB” and/or “I would prefer not to say” across waves (*n* = 192). No participant responded “I don’t know” at any wave. Those answering “I don’t identify with either of these” were excluded from the analysis (*n* = 59), as this category may have a distinct meaning to the respondent and not necessarily indicate belonging to any sexual minority group.

#### Covariates

Additional variables were included in the analysis to describe the study sample and/or to control for potential confounding variables or provide precise estimates of effect. Demographic indicators included age (in years), sex at birth (male or female), race/ethnicity (non-Hispanic White, non-Hispanic Black, Hispanic, Other), cohort (8th, 10th, or 12th grade at Wave 5), place of birth (i.e., United States, Other), county of residence (i.e., Travis, Bear, Dallas, Tarrant, Harris), and self-reported socioeconomic status (SES). To assess SES, participants were asked “In terms of income, what best describes your family’s standard of living in the home where you live most of the time?” and the response options provided were *“Very well off,” “Living comfortably,” “Just getting by,” “Nearly poor” and “Poor.” “Very well off”* was categorized as “High SES.” *“Living comfortably”* was categorized as “Middle SES” and *“Just getting by.”* Finally, *“Nearly poor”* and *“Poor”* were combined to derive “Low SES.” We also evaluated past 30-day use of combustible tobacco products (Yes/No), smokeless tobacco (Yes/No), and alcohol (Yes/No).

### Data Analysis

At baseline, descriptive statistics were generated for all variables. Chi-square tests were used to compare sociodemographic variables between the three subgroups (straight, LGB, mobile) according to participants’ sexual orientation. For comparing continuous variables, an analysis of variance (ANOVA) test was used (Table 1).

**Table 1.** Baseline characteristics of never ENDS^a^ users by sexual orientation (*n* = 1712)

Characteristic	Total n (%)	Straight (n = 1381) (%)	LGB^b^ (n = 139^1^) (%)	Mobile^c^ (n = 192) (%)	p value
**Age (mean, ±SD** ^d^)	16 (1.5)	16 (1.5)	15 (1.7)	16 (1.5)	**0.012**
**Prospective cohorts**					**0.002**
8th	551 (32)	32	44	25	
10th	617 (36)	37	26	35	
12th	544 (32)	31	30	40	
**Sex assigned at birth**					**<0.001**
Female	1006 (59)	56	75	69	
Male	706 (41)	44	25	31	
**Race/ethnicity**					0.268
White	529 (31)	31	37	29	
African American	259 (15)	16	9	14	
Latinx	619 (36)	36	37	37	
Other^e^	305 (18)	17	18	21	
**Self-reported SES** ^f^					**<0.001**
High	379 (22)	22	20	24	
Middle	1082 (63)	65	54	56	
Low	246 (15)	13	27	19	
**County of residence**					0.305
Harris (Houston)	388 (23)	22	24	24	
Bexar (San Antonio)	50 (3)	3	4	4	
Dallas (Dallas)	208 (12)	13	12	7	
Travis (Austin)	575 (33)	33	39	35	
Tarrant (Fort Worth)	491 (29)	29	22	30	

^a^ENDS: electronic nicotine delivery systems. Includes vape pens, e-hookah, hookah pens, MODS, tank systems, and e-cigars.

^b^Lesbian, gay, bisexual.

^c^Mobile: study participants who reported a combination of *“straight”* and/or *“LGB”* and/or *“I would prefer not to say”* across waves.

^d^Standard deviation.

^e^Includes Asian, *n* = 174; American Indian or Alaska Native, *n* = 21; Native Hawaiian or Other Pacific Islander, *n* = 10; and other (not listed), *n* = 100.

^f^Socio-economic status

**Figure 1.**
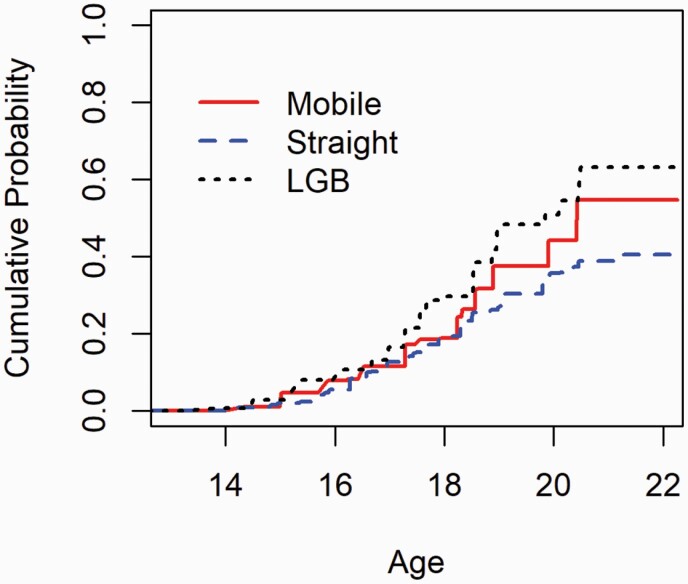
Cumulative probabilities of ever ENDS use by age and sexual orientation, among never ENDS users at baseline (*n* = 1712).

Nonparametric survival analyses for interval-censored data were implemented to estimate the hazards of ever ENDS initiation at different ages stratified by sexual orientation group (Figure 1). The generalized log-rank test was used to test the difference of the hazard of ever ENDS initiation among the sexual orientation groups.^33^

We used Cox proportional hazards regression with interval-censored data and the piecewise constant baseline hazard function to test for differences in the age of initiation of ever ENDS use by sexual orientation group, adjusting for sociodemographic variables: sex assigned at birth, race/ethnicity, cohort, and SES.^34^ The hazard ratio (HR) and its 95% CI are reported (Table 2). All analyses were conducted using SAS. Significance was set at *p* < 0.05, and 95% CIs were reported throughout the paper. All *p* values were two-sided.

**Table 2.** Differences in the age of initiation of ENDS use by sexual orientation.

Stratified variable	Description	HR^a^	95% CI^b^	
Unadjusted HR				
	Mobile^c^ vs. LGB^d^ (ref)	0.787	0.561	1.106
	Mobile vs. Straight (ref)	**1.362**	1.067	1.738
	LGB vs. Straight (ref)	**1.730**	1.315	2.274
Adjusted HR^e^				
	Mobile vs. LGB (ref)	0.956	0.677	1.349
	Mobile vs. Straight (ref)	**1.427**	1.116	1.828
	LGB vs. Straight (ref)	**1.494**	1.126	1.982

^a^Hazard ratio.

^b^Confidence intervals.

^c^Mobile: study participants who reported a combination of *“straight”* and/or *“LGB”* and/or *“I would prefer not to say”* across waves.

^d^Lesbian, gay, bisexual.

^e^Adjusted by race/ethnicity, sex at birth, cohort, and SES. However, only race/ethnicity is reported as it was the only significant variable in the model.

## Results

### Sample Characteristics

A total of 1712 individuals were included in the study sample (Table 1). At baseline (2016), the average age of participants was 16 years; at Wave 11 (2020), the average age was 19 years. Regarding birth-assigned sex, most of the study participants were female assigned (59%). The majority self-identified as Latinx (36%) and White (31%), followed by African Americans (15%) and other races and ethnicities (18%). Almost two-thirds of the individuals (63%) were classified as middle class. One-third of the participants were recruited in the city of Austin (33%). The rest of the sample was recruited in Fort Worth (29%), Houston (23%), Dallas (12%), and San Antonio (3%).

#### Sexual Orientation

A total of 1381 (81%) of study participants consistently self-identified as Straight individuals, 139 (8%) consistently self-identified as LGB individuals, and 192 (11%) were categorized as mobile individuals whose sexual orientation changed across waves. Among mobile group, about 56% of participants moved from Straight to LGB and 13% moved from LGB to straight, while the rest of respondents in this group changed their response three or more times during the five points of data collection. Mobile and straight individuals were slightly older than LGB individuals (*p* = 0.012), and a larger proportion of mobile and LGB individuals were assigned female at birth compared to Straight individuals (*p* < 0.001). Across the groups (straight, LGB, mobile), statistical significance was reported for cohort (*p* = 0.002) and self-reported SES (*p* < 0.001).

#### ENDS Initiation

By age 18, 19% of Straight individuals, 30% of LGB individuals, and 19% of mobile individuals reported ENDS initiation (*p* < 0.001). The hazard plot (Figure 1) shows that compared to straight youth, the hazard of ever ENDS initiation peaked earlier for LGB individuals (HR = 1.73, 95% CI: 1.32 to 2.27) and mobile individuals (HR = 1.36, 95% CI: 1.07 to 1.74) throughout the whole study period, in unadjusted models. The null hypothesis (ENDS initiation’s hazards are equal across the groups: straight, LGB, mobile) was rejected (*p* < 0.001), and these differences between groups increased over the observation period, from 13 years old through the age of 22.

#### Use of Other Tobacco Products and Alcohol Consumption

Since very few of these individuals reported past 30-day use of combustible tobacco products (0.4%), past 30-day use of smokeless tobacco (0.2%), and past 30-day alcohol consumption (8%) at baseline (Wave 5), these variables were not included in further analyses.

#### Potential Predictors of ENDS Initiation

The results from the Cox regression analysis showed that after adjusting for sex assigned at birth, race/ethnicity, prospective cohort, and SES, the onset of ENDS use occurred at earlier ages among mobile individuals (HR = 1.43, 95% CI: 1.12 to 1.83) and LGB individuals (HR = 1.49, 95% CI: 1.13 to 1.98), compared to straight adolescents.

## Discussion

Since 2014, ENDS have become the most commonly used tobacco product reported by youth, surpassing traditional cigarette use.^35^ Previous reports assessing the prevalence of ever ENDS use among youth have indicated that those identified as lesbian, gay, bisexual, and queer/questioning youth vape at higher rates than their heterosexual counterparts.^6,13,36,37^ In the current study, we expanded upon the existing scientific literature by examining ENDS initiation prospectively to account for changes in individuals’ patterns of sexual orientation over time.

Our work provides evidence that over the study period and compared to straight subjects, the hazard of becoming an ENDS user peaked earlier not only for LGB adolescents and youth, but also for mobile individuals who experienced at least one change in sexual orientation over time. Further, this risk difference was statistically significant even after accounting for potential confounders. Findings of this research effort shed light on the potential misclassification of at-risk populations when sexual orientation data is not regularly collected and accurately recorded, which ultimately may underestimate the true risk among mobile individuals, but also negatively impact the effectiveness of any ENDS use prevention program among adolescents and young adults who are particularly prone to experience changes in their sexual orientation (e.g., attraction, behavior, and identity) through adulthood.

In our sample, while 81% of the study participants consistently self-identified as Straight individuals (81%) and 8% as LGB youth, 11% prospectively reported sexual orientation mobility across waves. This clearly indicates that defining sexual minority groups based on a single measure of sexual orientation at one point in time may lead to misidentification and underreporting. For research purposes, placing the sexual orientation question around behavior items rather than among demographics could increase data collection accuracy and decrease response bias.^38^ However, further testing should be conducted among adolescents and youth to determine best practices for placing, structuring, and wording sexual orientation and gender identity questions; understanding how these items perform; and determining levels of nonresponse across subgroups. In any case, routinely documenting sexual orientation at every available opportunity (e.g., study assessments, clinical visits, etc.) seems to be a necessary first step to lay the ground work for effectively identifying vulnerable groups, so as to increase the effectiveness of tobacco prevention and control programs.

Research has begun to study differences in certain health conditions by individuals’ changes in same-sex or other-sex desire across both short-term and long-term periods.^20,39,40^ In a cross-sectional study conducted among adult individuals, shifts in the directionality of sexual orientation during the lifetime were significantly associated with adverse mental health outcomes such as stress, anxiety, and depression.^41^ In a cohort study from a nationally representative sample, changing sexual orientation across waves was associated with cigarette smoking initiation and current smoking.^42^ While there is compelling evidence of the association between mental health problems and ENDS use among adolescents and young adults,^43,44^ sexual orientation changes over time in this particular vulnerable population is not well-established. Therefore, sexual orientation mobility may present more of a challenge for ENDS prevention and control programs targeting youth.

A web-based search conducted in 2020 by Liu et al. identified eight ENDS prevention programs, seven ENDS cessation programs, and one program that addressed both ENDS prevention and cessation among adolescents. Most of these programs have shown promising results at changing adolescent perceptions and behavior about e-cigarette use.^45^ However, the effectiveness of these programs among SGM groups remains unknown. In 2016, the US Food and Drug Administration (FDA) launched *“This Free Life”* campaign through out-of-home advertising (e.g., billboards, print media, and events), social media platforms and influencers to prevent and reduce tobacco consumption among SGM adults aged 18–24.^46^ This large-scale public educational campaign proved to elevate the awareness of the health damage caused by tobacco products, including ENDS, among SGM young adults. However, similar educational efforts for SGM adolescents transitioning into young adulthood are lacking. Therefore, our findings provide relevant information that can inform the design of evidence-based interventions targeting SGM teens and youth.

However, this study had a few limitations. We did not consider ENDS use pattern (daily vs. nondaily use) or distinguish between different types of ENDS products (e.g., cig-a-like, vape pen, pod, mod, among others)^47^ in this analysis, given limited sample size. Therefore, differences in ENDS use patterns after initiation and product type might differ by sexual orientation. This should be explored in future research. Due to a lack of sexual orientation data collection in the TATAMS study between Waves 1 and 4, our sample was limited to respondents participating in Wave 5 or later waves. Also, we did not include in the survival models past use of combustible tobacco products, smokeless tobacco, or past 30-day alcohol consumption due to low reporting among study participants at baseline.

Given that most ENDS users are at higher risk of becoming multiple tobacco users (ENDS users who simultaneously use any other tobacco product) and that multiple tobacco use may reinforce nicotine addiction,^48^ it is important to investigate further the link of these behaviors with ENDS use uptake and/or progression, especially by sexual orientation. In addition, findings are limited to adolescents in Texas and may not generalize to other geographic locations. While the population in Texas has become increasingly urban, a comprehensive national report indicates that between 15% and 20% of sexual and gender minority groups in the US live in rural areas. These individuals may experience discrimination and stress substantially differently from their urban counterparts due to “the [relative] lack of community, resource, and services in rural areas.” ^49^ Our results are also limited to LGB adolescents and youth, not necessarily encompassing all sexual minority groups with a non-heterosexual sexual orientation. Also, future longitudinal studies with larger samples are needed to better differentiate the directionality of sexual orientation mobility among adolescents and youth, as well as the timing, frequency, and duration of those changes over the observation period. Finally, there could be significant intragroup differences in early ENDS initiation among study participants categorized in the LGB group, indicating the need for enhanced research efforts with large study samples that broaden the possible data range and form a better analysis picture for ENDS initiation among lesbian, bisexual female, gay, and bisexual male adolescents and youth.

Notwithstanding the above limitations, to the best of our knowledge, this is the first study examining whether ENDS initiation differs across subgroups defined by their sexual orientation- straight, LGB, and mobile individuals- in a contemporary sample of adolescents (13–22 years of age) over time (2016-2020). Understanding the implications of sexual orientation mobility on early-onset of ENDS use will be critical for developing inclusive public health programs aimed at preventing or delaying ENDS use and for providing practical recommendations at state and local levels.

## Supplementary Material

A Contributorship Form detailing each author’s specific involvement with this content, as well as any supplementary data, are available online at https://academic.oup.com/ntr.

Since the data collection process is still ongoing, research data from The Texas Adolescent Tobacco and Marketing Surveillance Study (TATAMS) 2.0 cannot be shared.

ntab181_suppl_Supplementary_Taxnomy_Form
